# A virtual bridge to Universal Healthcare in India

**DOI:** 10.1038/s43856-022-00211-7

**Published:** 2022-11-16

**Authors:** Biswanath Ghosh Dastidar, Shailesh Suri, Vikranth H. Nagaraja, Anant Jani

**Affiliations:** 1 GD Institute for Fertility Research, Kolkata, 700025 India; 2 Cambridge University Hospitals, Cambridge, CB20QQ UK; 3 Oxford India Centre for Sustainable Development, University of Oxford, Oxford, OX2 6HD UK; 4 Department of Engineering Science, Institute of Biomedical Engineering, University of Oxford, Oxford, OX1 3PJ UK; 5 Oxford Martin School, University of Oxford, Oxford, OX1 3BD UK

**Keywords:** Epidemiology, Health services

## Abstract

Dastidar et al. discuss how recent policy measures, and favourable technological and infrastructural landscapes are bolstering India’s ability to implement virtual care. These developments were accelerated by the need for an alternative model of care during the COVID-19 lockdowns and may provide a bridge to Universal Healthcare in India.

## Teaser

Virtual care could be instrumental in helping India achieve Universal Healthcare. COVID-19 has catalysed virtual-care adoption by practitioners and end-users alike. Recent inclusive policy measures, a favourably-evolving technological landscape, and infrastructural development empower India to technology-leapfrog existing Western healthcare systems.

India went into COVID-19 lockdown on 23 March 2020, resulting in widespread disruption of routine healthcare. This led to significant task-shifting in hospitals, changing inpatient to outpatient care, and outpatient to telemedicine-driven home care. Consequently, in-person appointments went down by 32%, and online consultations went up by 300%^1^. Within six months of launch, one of India’s largest private-sector healthcare providers, Apollo Hospital group’s digital consultation platform (Apollo 24/7), enroled four million people with about 30,000 downloads per day^2^. Fifty million citizens accessed healthcare online from March–May 2020, 80% of whom were first-time users^2^.

As another example, on 29 March 2020, within a week of the first COVID-19 lockdown, one of the study co-authors (BGD) helped launch a voluntary pan-India telecare initiative to offer free teleconsultations to anyone unable to access in-person care. Thirty specialist physicians across eight cities treated over 500 patients. The majority had minor ailments which were resolved online; in-person consultations were arranged to treat four patients; and one gentleman was referred to a medical college where he underwent life-saving surgery. These physicians were telecare novices operating out of a hastily-formed Facebook page for outreach. But this experience led to individual and collective reflection upon the potential of virtual care in India, scaled up and supported by the right tools.

This article discusses lessons learned from this switch to virtual care and proposes steps that may help India leverage virtual healthcare to deliver on its commitment to provide Universal Healthcare (UHC) to its citizens within a largely threadbare public health infrastructure.

## India’s healthcare challenge

India’s healthcare expenditure is consistently amongst the world’s lowest at 1.28% of the gross domestic product (GDP)^3^. Though public hospitals provide free care, the quality is highly variable, from internationally-acclaimed centres to unacceptably low-quality underfunded facilities that face frequent staff and equipment shortages^4^. Besides, many unregulated clinics exist, and only nine out of 28 states mandate physician participation in Continuing Medical Education^5^.

Rural outposts and urban slums have typically remained underserved, and while general access to healthcare is heterogeneous within and between states, the starkest variation is seen between rural and urban areas. Although 70% of India’s population lives in villages, 60% of hospitals, 75% of pharmacies, and 80% of physicians are based in cities and suburbs, with the number of physicians per capita in rural areas being 25% of those in urban settings^6^. The scarcity of healthcare infrastructure in remote villages often necessitates travel for nearly a day or more to access primary care from qualified providers.

Thus, many individuals either forego needed care or rely on out-of-pocket payments for care, accounting for almost 62.6% of total health expenditure—one of the highest globally^7^. Unsurprisingly, healthcare costs are a considerable driver of poverty in India^6^, of which extreme poverty (i.e., subsistence on less than $1.9/day) dropped by a mere 12% over 8 years from 2011–2019, as compared to 17.5% over the previous 7 years from 2004–2011^8^. Indeed, any strategy by India to haul its massive population out of poverty must necessarily include measures to provide UHC nationwide.

## India’s Universal Healthcare ambitions

Inspired by UHC models across the world, India launched the Ayushman Bharat insurance scheme in 2018, which aims to provide cashless secondary and tertiary healthcare to the economically disadvantaged at participating hospitals registered with the scheme as a first step towards delivering UHC by 2030^9^.

Delivering UHC in India is made more challenging by India’s shifting and increasing burden of non-communicable diseases. Being amidst an epidemiologic transition linked to risk factors like poor diet, smoking, and pollution, recent decades have witnessed a rising incidence of cardiovascular diseases, diabetes, and cancer; with one study documenting a 13.4% cumulative incidence of Type-2 diabetes (20.2 per 1000 person-years)^10^—significantly more than in the US (7.6 per 1000 person-years)^11^. These diseases alone are projected to cost India approximately $2.3 trillion from now through 2030—1.5 times India’s GDP and about 35 times India’s total annual health expenditure^12^. Given India’s inadequate health spending and infrastructure, combined with increasing need, UHC delivery solely through traditional pathways entailing in-person interactions at brick-and-mortar institutions seems hardly a tenable option.

Given the above constraints, we propose that adopting a novel virtual-physical bridge wherein virtual care is offered as the default first step in the care pathway before upstream escalation, or bridging, to in-person patient management when deemed necessary could be an effective new standard of care for delivering UHC in India.

## Readiness for virtual care

India is well-positioned to adopt virtual care at a population level, being amongst the fastest-growing digital consumer markets with over 0.5 billion internet subscribers using more than 8 GB/month and some of the lowest data costs globally^13^. At a policy level, the government released the Telemedicine Practice Guidelines in March 2020 to eliminate uncertainty about telemedicine’s legitimacy, serve as a comprehensive resource, and provide a robust medicolegal framework for patients and practitioners alike. At an infrastructural level, the Ayushman Bharat Digital Mission (ABDM), launched in 2021, established a digital ecosystem to enable data access and sharing (medical history, investigations, prescriptions, etc.) between patients and healthcare providers, leveraging a unique national health identifier for each citizen—Ayushman Bharat Health Account (ABHA); and regulated by a central consent manager. The platform further aggregates digital registers of healthcare organisations and professionals nationally.

## Practise of virtual care

India’s National Telemedicine Service—eSanjeevani, a first-of-its-kind free telemedicine initiative by any country—was established by the Ministry of Health & Family Welfare in 2020. eSanjeevani comprises two key pillars: eSanjeevani Ayushman Bharat–Health and Wellness Centre (AB–HWC) and eSanjeevaniOPD (Outpatient Department). eSanjeevani AB–HWC, based on a hub-and-spoke model, facilitates a real-time referral-to-prescription loop by enabling provider-provider communication between a paramedic or general practitioner sitting with a patient at the HWC (spoke) and a specialist at a tertiary referral hospital (hub). First piloted in November 2019 in Andhra Pradesh, eSanjeevani AB–HWC has been implemented in over 25,000 hubs and about 100,000 spokes nationwide. eSanjeevaniOPD facilitates medical teleconsultations from the confines of patients’ homes. Rolled out in April 2020 as a mobile app, it has seen over three million downloads and completed over 30 million teleconsultations.

While these are initial steps, there appears to be a genuine promise for telemedicine-led virtual care transformation in India.

## Steps toward virtual care transformation

Not all areas of healthcare will be amenable to delivery through virtual routes, but for those that are, facilitating a virtual care transformation in India will require some key steps.

At a systems level, novel regulatory and governance approaches are required to address issues of consent, data security, and confidentiality and ensure that risks of virtual care are mitigated while designing innovations such as health apps and central data repositories. Procurement policies must provide value for money, i.e., delivering good patient outcomes while optimising resource utilisation. To ensure equity and prevent digital exclusion, citizens need to be supported to transition to the realities of virtual care through education in schools and purpose-built community centres, particularly in villages and urban/semi-urban slums.

From a care delivery perspective, clinical areas particularly suited to digital delivery must be identified. Standardised operating protocols (SOPs) must be developed in consultation with key stakeholders in the care pathway (patients, physicians, nurses, paramedics, and policy-makers) to guide digital practice in these areas and optimise risk mitigation. Healthcare professionals need to be trained on how to integrate virtual care within established care pathways while also introducing novel telemedicine-led care. To protect against digital exclusion, strategic triaging protocols must ensure patients are not deprived of high-quality care, whether virtual or in-person, while minimising unnecessary referrals for in-person care. Continuity of care must be guaranteed by regularly updating ABHA-linked patient medical records on the ABDM platform.

Finally, a digitally-savvy workforce of healthcare professionals must be trained, leveraging modified curricula which address risks, pitfalls and governance issues associated with digital care delivery. Most importantly, such training must also be imparted to create a cadre of paramedics and allied health professionals, looking beyond the Indian health education system’s traditionally exclusive focus on training medical and nursing practitioners. Virtual care transformation can only succeed once such a workforce is ready to facilitate last-mile care delivery. Of particular relevance would be an efficient and digitally-empowered midwifery workforce. It is estimated that universal coverage of midwife-led care could avert 67% of maternal deaths, 64% of neonatal deaths, and 65% of stillbirths, besides providing 90% of all sexual, reproductive, maternal, newborn, and adolescent health services^14^.

## Commitment to Universal Healthcare

India could have handled the initial waves of the COVID-19 pandemic better, but hope is not lost. The recent announcement of a $6.8 billion commitment to improve healthcare infrastructure could be an essential step toward strengthening virtual-care capacity^15^. Our proposed measures may prove to be crucial investments through which India can continue leveraging virtual care more innovatively, efficiently, and effectively on its journey toward UHC and potentially serve as a ready blueprint for other developing countries.

### Reporting summary

Further information on research design is available in the Nature Portfolio Reporting Summary linked to this article.

## Supplementary information

Reporting Summary
